# Symmetry breaking by enzyme-catalyzed epoxide hydrolysis

**DOI:** 10.1107/S2052252518007005

**Published:** 2018-05-18

**Authors:** Christopher J. Schofield

**Affiliations:** a Chemistry Research Laboratory, University of Oxford, Mansfield Road, Oxford OX1 3TA, UK

**Keywords:** epoxide hydrolase, stereoselectivity, biocatalysis

## Abstract

Janfalk Carlsson *et al.* [*IUCrJ* (2018), **5**, 269–282] describe studies on factors determining selective ring opening of methylstyrene oxide stereoisomers by the epoxide hydrolase StEH1. The stereo-differentiating step is selective hydrolysis of an alkylated intermediate formed by reaction of the epoxide with an aspartyl residue.

The strained epoxide ring is important in organic synthesis, because of its reactions with nucleophiles and ability to be converted to other functional groups of importance in the synthesis of biologically active compounds. Understanding the stereoselectivity of epoxide formation and ring opening is therefore of considerable interest. Work on the formation of chiral epoxides and their application was massively stimulated by the invention of the Sharpless asymmetric epoxidation reaction (Katsuki & Sharpless, 1980 ▸). However, detailed mechanistic studies on stereo- and regiochemical aspects of their ring opening are perhaps less well travelled. In a recent article in **IUCrJ**, Janfalk Carlsson *et al.* (2018 ▸) reveal studies on the factors regulating the enzyme-catalyzed ring opening of methyl­styrene oxide by the epoxide hydrolase StEH1. They coupled mutagenesis and structural studies with detailed kinetic analyses, employing both steady-state and pre-steady-state methods, and modelling to give a detailed picture of the factors regulating the product selectivities obtained with the (*S*,*S*)- and (*R*,*R*)-methylstyrene oxide substrates. The mechanism of StEH1 proceeds *via* a covalent intermediate formed by reaction of an active-site aspartyl residue (Asp105) with one of the epoxide carbons to give an enzyme-bound alkoxide intermediate stabilized by hydrogen bonding with the phenol groups of tyrosine residues (Fig. 1 ▸). Subsequent ester hydrolysis yields the vicinal diol product. Thus, the reaction proceeds with stereochemical inversion at the epoxide carbon, which is attached by the nucleophilic Asp105. However, the reaction is complicated because attack can occur at either of the two epoxide carbons.

The results of Janfalk Carlsson *et al.* elegantly rationalize an interesting experimental observation that has not been adequately explained before, *i.e.* that the (*S*,*S*)-epoxide substrate reacts to give only the (1*R*,2*S*)-diol product, whereas the (*R*,*R*)-epoxide gives a mixture of (1*R*,2*S*)- and (1*S*, 2*R*)-enantiomers. The authors show that the (*R*,*R*)-epoxide can react *via* ring opening at either of the epoxide carbons to give alkoxide intermediates which are both hydrolysed, so giving an enaniomeric mixture of products. By contrast, whilst the (*S*,*S*)-epoxide also reacts *via* both epoxide carbons, only the pro-(*R,S*)-alkoxide enzyme intermediate reacts productively to give the stereochemically pure (1*R*,2*S*)-diol product.

The work thus reveals the importance of combining structural analyses with detailed kinetic analyses in solution. Augmented by modelling studies, enabled by contemporary computing power, such *integrated approaches* to understanding enzyme, and non-enzyme, catalysis has enormous potential for identifying a new generation of protein-based and other catalysts, including with non-biological functional groups, aimed at solving major societal problems.

## Figures and Tables

**Figure 1:**
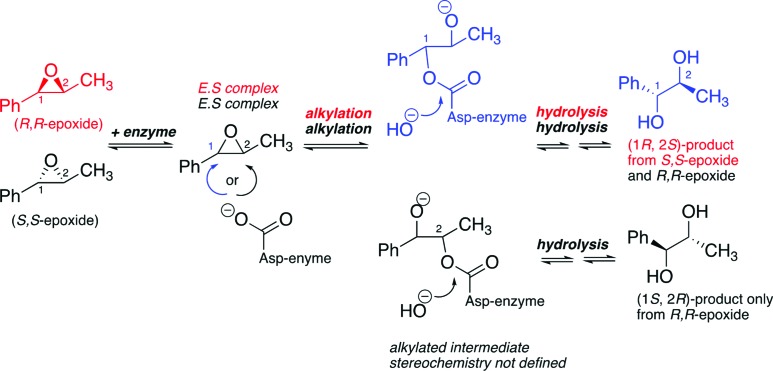
Whereas reaction of the (*S*,*S*)-epoxide gives only the (1*R*,2*S*)-diol product, the (*R*,*R*)-epoxide gives both (1*R*,2*S)*- and (1*S*,2*R)*-products. The key product differentiating step in the case of the (*S*,*S*)-epoxide is selective hydrolysis of the alkyl­ated intermediate formed by reaction of the (*S*,*S*)-epoxide at C-1 over that formed by reaction at C-2. Note the stereochemistry of the alkylated intermediates depends on that of the starting epoxide (there are two possible isomers of the alkylated intermediate for each substrate).
